# Publications Received

**Published:** 1859-02

**Authors:** 


*Publications Received.*—We have received from Messrs. Blanchard & Lea, of Philadelphia, Dalton's Treatise on Human Physiology, Ricord and Hunter on the Venereal by Bumstead, Condie on Diseases of Children, and Erichsen's Science and Art of Surgery; from Wiley & Halsted, Green on Bronchitis, and Prescriptions of American Practitioners by the same author; from Robert M. De Witt, New York, Lectures on Obstetrics, Tyler Smith. Edited by A. K. Gardner; from J. B. Lippincott & Co., Malgaine's Treatise on Fractures, Translated by Packard.

We have also received the eleventh volume of the Transactions of the American Medical Association. This has been delayed this year, not from any negligence of the publishing committee, but owing to the difficulty in receiving some of the proofs. These publications will receive an early attention. We have also quite a number of pamphlets on our table which we hope to be able to notice in our next issue.

